# Prognostic Role of Dynamic Changes in Serological Markers in Metastatic Hormone Naïve Prostate Cancer

**DOI:** 10.3390/cancers15174392

**Published:** 2023-09-02

**Authors:** Soumyajit Roy, Yilun Sun, Christopher J. D. Wallis, Amar U. Kishan, Scott C. Morgan, Daniel E. Spratt, Shawn Malone, Fred Saad

**Affiliations:** 1 Department of Radiation Oncology, Rush University Medical Center, Chicago, IL 60612, USA; 2 Department of Epidemiology, Usher Institute, The Edinburgh University, Edinburgh EH16 4SS, UK; 3 Department of Population and Quantitative Health Sciences, School of Medicine, Case Western Reserve University, Cleveland, OH 44106, USA; 4 Department of Urology, Mount Sinai Hospital and University Health Network, University of Toronto, Toronto, ON M5G 1X5, Canada; wallis.cjd@gmail.com; 5 Department of Radiation Oncology, University of California Los Angeles, Los Angeles, CA 90095, USA; aukishan@mednet.ucla.edu; 6 Division of Radiation Oncology, The Ottawa Hospital Cancer Centre, University of Ottawa, Ottawa, ON K1H 8L6, Canada; smorgan@toh.ca (S.C.M.); smalone@toh.ca (S.M.); 7 Department of Radiation Oncology, University Hospital Seidman Cancer Center, Case Western Reserve University, Cleveland, OH 44106, USA; 8 Department of Surgery, Université de Montréal, Montreal, QC H2X 0A9, Canada

**Keywords:** metastatic hormone sensitive prostate cancer, prostate-specific antigen, hemoglobin, neutrophil to lymphocyte ratio, joint model

## Abstract

**Simple Summary:**

In this exploratory analysis of a randomized controlled trial, we found that dynamic changes in simple laboratory-based markers such as prostate-specific antigen (PSA) could be used to predict survival in patients with metastatic prostate cancer that is sensitive to hormonal manipulation. We developed a model that captures information on dynamic changes in PSA along with hemoglobin (Hb), neutrophil to lymphocyte ratio (NLR), platelet to lymphocyte ratio (PLR), and lymphocyte to monocyte ratio (LMR), and this model was found to be clinically more useful compared to the “treat all” strategy. This model could be used to design future adaptive trials that will investigate sequential treatment personalization in metastatic hormone sensitive prostate cancer patients.

**Abstract:**

We investigated whether inter-patient variation in the dynamic trajectory of hemoglobin (Hb), neutrophil to lymphocyte ratio (NLR), platelet to lymphocyte ratio (PLR), lymphocyte to monocyte ratio (LMR), and prostate-specific antigen (PSA) can prognosticate overall survival (OS) in de novo mHSPC. This is a secondary analysis of the LATITUDE trial in which high-risk de novo mHSPC patients were randomly assigned to receive either androgen deprivation therapy (ADT) plus abiraterone or ADT plus placebo. We used a five-fold cross-validated joint model approach to determine the association of temporal changes in the serological markers with OS. Decision curve analysis was applied to determine the net benefit. When dynamic changes in Hb, LMR, NLR, PLR, and PSA were included in a multivariate joint model, an increase in the log of the current value of PSA (HR: 1.24 [1.20–1.28]) was associated with inferior OS. A multivariate joint model that captured dynamic trajectory of Hb, NLR, PLR, LMR, and PSA up to 24 months, showed a net benefit over the “treat all” strategy at a threshold of probability of approximately ≥30% while no net benefit was seen when dynamic change in PSA was omitted. Our joint model could be used for designing future adaptive trials investigating sequential treatment personalization.

## 1. Introduction

Despite significant advancements in management strategies in the last decade, patients with newly diagnosed metastatic prostate cancer are at a significant risk of mortality [1,2,3,4,5,6,7,8,9,10]. Although considered as a single entity, metastatic hormone sensitive prostate cancer (mHSPC) is essentially a spectrum of disease with varying clinical presentation, tumor biology, and overall prognosis [11,12]. Despite this underlying heterogeneity in underlying biology and treatment strategies, prostate-specific antigen (PSA) has been consistently found to be an indicator of underlying cancer activity and confers important prognostic information [13,14,15,16].

Additional efforts in exploring the underlying biology have shown chronic inflammation to be a driver of disease progression and metastasis in prostate cancer [17,18]. Several studies have investigated the predictive and prognostic value of serological markers of inflammation in metastatic castrate resistant prostate cancer (mCRPC) [19,20,21]. For example, in a small retrospective Japanese study, baseline neutrophil to lymphocyte ratio (NLR) level was an independent predictor of overall survival (OS) in mCRPC treated with abiraterone [22]. In a systematic review and meta-analysis of patients with mCRPC, elevated NLR and platelet to lymphocyte ratio (PLR) had a significant association with increased risk of mortality in mCRPC patients treated with abiraterone and enzalutamide [21]. In contrast, in a secondary analysis of the COU-AA-302 trial, a treatment-induced change in the NLR level from baseline had no association with OS [23].

The prognostic value of the serological markers of inflammation has not been well studied in patients with mHSPC. In a large population-based study, higher baseline NLR or PLR and lower baseline hemoglobin (Hb) were associated with increased risk of mortality [24]. Similarly, prior work has demonstrated the prognostic association of baseline hemoglobin and PSA with OS in de novo mHSPC [25,26,27]. However, given the inherent limitation of static baseline prognostic models [28], they fail to show the real-time association of survival with dynamic changes in these markers over time, which potentially reflects a combined time- and treatment-dependent variation in the underlying disease process among patients [29,30]. We performed a secondary analysis of the LATITUDE study to determine whether inter-patient variations in the dynamic trajectory in serological markers such as Hb, NLR, PLR, lymphocyte to monocyte ratio (LMR), and PSA over time could prognosticate OS and prostate cancer-related survival (PCSS) in men with de novo mHSPC.

## 2. Patients and Methods

LATITUDE was an international, multicentric, phase III randomized controlled study (NCT01715285) conducted at 234 clinical sites across 34 countries [10]. In this trial, men with high-risk mHSPC (defined as 2 or more of the following: ≥3 bony lesions, visceral metastasis, and Gleason score ≥ 8) were randomly allocated to receive either androgen deprivation therapy (ADT) plus abiraterone acetate plus prednisone (the abiraterone plus ADT group) or ADT plus dual placebos (the ADT alone group). Study medications were administered continuously in 28-day cycles. Laboratory parameters were monitored at each clinic visit, which was monthly for the first year and every alternate month thereafter. Detailed randomization procedures including co-primary endpoints (i.e., radiographic PFS and OS) have been reported previously [10,31].

## 3. Statistical Analyses

The objective of this study was to determine whether inter-patient variation in the trajectories of dynamic changes in Hb, NLR, PLR, and LMR prognosticate primarily for OS and PCSS with or without inter-patient variation in the trajectory of dynamic change in PSA. We included patients with a baseline measurement and at least two post-baseline measurements of these laboratory-based markers. To capture the independent association between the dynamic change in the markers of each patient and the hazard of the outcomes (OS and PCSS), we utilized the framework of joint modeling for longitudinal and survival outcomes [32].

For all the markers, we applied separate five-fold cross-validated univariate joint models to determine the association of dynamic changes in these markers with OS and PCSS. A Cox proportional hazard regression model was constructed for the time-to-event sub-model that included Eastern Cooperative Group (ECOG) performance status; number of skeletal lesions (0–9 vs. ≥10); presence of liver metastasis; nodal stage (N0/Nx vs. N1); Gleason score (<9 vs. 9–10); baseline Hb; log of baseline PSA, which was created by logarithmic transformation after addition of one to all baseline PSA values; worst pain score at baseline; and treatment arm. A linear mixed-effects model was built for the longitudinal sub-model with an interaction term for treatment arm and time of evaluation in addition to fixed covariables: treatment arm, time of evaluation, and baseline value of the respective marker. Time of assessment was included as random slope while patients were included as random intercepts in the mixed models. The two sub-models were linked through a shared random effect—often referred to as a current value association structure since it assumes that the log hazard of the event at time *t* is linearly associated with the value of the longitudinal sub-model’s linear predictor also evaluated at time *t*.

Subsequently, separate five-fold cross-validated multivariate joint models with horseshoe regularization were constructed to determine the association of the trajectory of dynamic changes in Hb, NLR, PLR, and LMR with OS and PCSS. Thereafter, we built an additional five5-fold cross-validated multivariate joint model with horseshoe regularization to explore the association of trajectory of dynamic changes in the aforementioned markers (Hb, NLR, PLR, and LMR) along with dynamic changes in PSA with OS and PCSS, respectively. PSA values were converted to log scale after adding one to the respective values. The calibration of the joint models was checked by the integrated calibration index (ICI), E50, and E90 [33]. The ICI is defined as the weighted average absolute difference between observed and predicted probabilities. E50 denoted the median absolute difference between observed and predicted probabilities, while E90 denoted the 90th percentile of this absolute difference. Time-varying Brier scores were reported as a measure of discriminative index of the models by capturing longitudinal data until 12 months or 24 months and then subsequent follow-up up to 60 months. Lower ICI, E50, and E90 indicate superior model calibration. A Brier score represents the average squared distance between the observed survival status and the predicted survival probability and is always a number between zero and one, with zero being the best possible value. Given that these are right censored data, the score was adjusted by weighing the squared distances using the inverse probability of censoring weights method. To determine the clinical utility of the multivariate joint models with longitudinal information up to 12 months and 24 months, we applied decision curve analysis (additional details in Supplementary Methods) [34,35]. We reported 95% confidence intervals with two-sided *p* < 0.004 being set as a threshold for significance. All statistical analyses were performed using R version 3.6.3 with its packages.

## 4. Results

Overall, 1199 patients were randomly allocated to one of the two treatment arms in the LATITUDE study. Information on all baseline characteristics was available in 1194 patients. Overall, 1138 patients had baseline measurement of all laboratory markers with at least two post-baseline measurements. They were eligible for this secondary analysis. Overall, 563 were assigned to the abiraterone plus ADT arm while 575 belonged to the ADT alone group. There was no statistically significant difference in the distribution of baseline characteristics between the two treatment groups (Table 1). The median number of assessments in the study cohort was 25 (IQR: 16–35). Median follow-up for surviving patients was 52.3 months (IQR: 51.6–53.5) for the abiraterone arm and 51 months (IQR: 50–52) for the ADT alone arm.

On univariate joint model for OS, a 1 g/dL increase in the current value of Hb was associated with superior OS (hazard ratio (HR): 0.77; 95% confidence interval (CI): 0.73–0.82; *p* < 0.001). Similarly, every increase the current value of LMR by five points was associated with significantly superior OS (HR: 0.53; 95% CI: 0.41–0.69; *p* < 0.001). In contrast, every increase in the current value of PLR by 100 points (HR: 1.60; 95% CI: 1.42–1.80; *p* < 0.001) and every increase in the current value of NLR by one point (HR: 1.29; 95% CI: 1.21–1.38; *p* < 0.001) was associated with significantly inferior OS. On univariate joint modelling for PCSS, every 1 g/dL increase in the current value of Hb (HR: 0.77; 95% CI: 0.72–0.83; *p* < 0.001) and every five points increase in the current value of LMR (HR: 0.52; 95% CI: 0.37–0.71; *p* < 0.001) was associated with significantly superior PCSS, while every 100 points increase in PLR (HR: 1.61; 95% CI: 1.41–1.83) and every point increase in NLR (HR: 1.27; 95% CI: 1.17–1.37; *p* < 0.001) was associated with significantly inferior PCSS. The calibration and discrimination indices of the univariate joint models (from five-fold cross-validation) have been summarized in the Supplementary Material (Supplementary Figures S1–S16).

**Figure 1:**
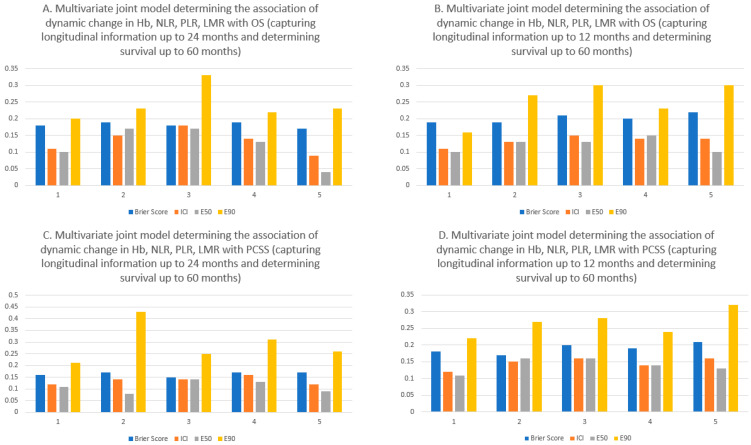
Summary of the time-varying Brier scores, integrated calibration index (ICI), E50, and E90 of 5-fold cross-validated multivariate joint model determining the association of dynamic change in Hb, NLR, PLR, and LMR with overall survival and prostate cancer-specific survival. The top panel shows the calibration indices and the time-varying Brier scores for overall survival (using dynamic longitudinal information up to 24 months (**A**) and 12 months (**B**)) while the bottom panel shows the calibration indices and the time-varying Brier scores for prostate cancer-specific survival (using dynamic longitudinal information up to 24 months (**C**) and 12 months (**D**)). A lower Brier score indicates superior discriminative power of the model and similarly lower ICI, E50, and E90 indicate superior calibration (i.e., less difference between observed and predicted probabilities).

On the multivariate joint model for OS, when the dynamic changes in Hb, NLR, PLR, and LMR were combined, every 1 g/dL increase in the current value of Hb was associated with significantly superior OS (HR: 0.80; 95% CI: 0.75–0.86; *p* < 0.001) and every point increase in NLR was associated with significantly inferior OS (HR: 1.19; 95% CI: 1.06–1.33; *p* = 0.003) (Table 2). On multivariate joint modelling for PCSS, every 1 g/dL increase in the current value of Hb was associated with significantly superior PCSS (HR: 0.81; 95% CI: 0.75–0.87; *p* < 0.001), while there was no significant association between the dynamic change in NLR with PCSS (HR: 1.11; 95% CI: 0.97–1.27; *p* = 0.13) (Table 2). The time-varying Brier scores from the five-fold cross-validated joint model for OS ranged from 0.17 to 0.19 and 0.19 to 0.22 with longitudinal information up to 24 months and 12 months, respectively (Figure 1). The time-varying Brier scores from the five-fold cross-validated joint model for PCSS ranged from 0.15 to 0.17 and 0.17 to 0.21, with longitudinal information up to 24 months and 12 months, respectively (Figure 1).

**Figure 2:**
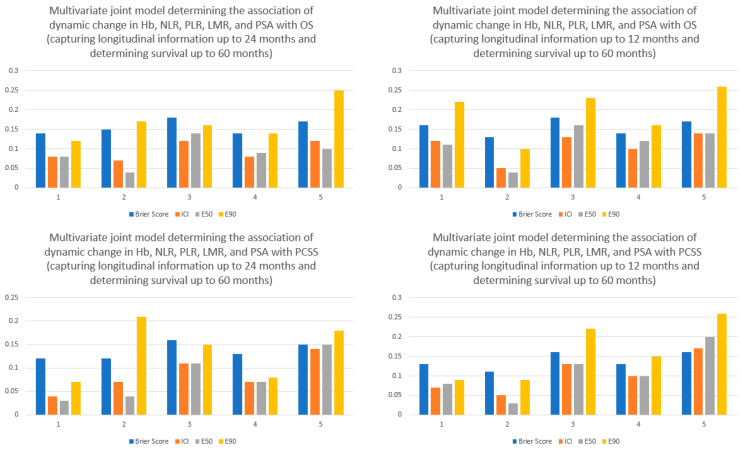
Summary of the time-varying Brier scores, integrated calibration index (ICI), E50, and E90 of five-fold cross-validated multivariate joint model determining the association of dynamic change in Hb, NLR, PLR, LMR, and PSA with overall survival and prostate cancer-specific survival. The top panel shows the calibration indices and the time-varying Brier scores for overall survival (using dynamic longitudinal information up to 24 months ((**Top left**) and 12 months (**Top right**)) while the bottom panel shows the calibration indices and the time-varying Brier scores for prostate cancer-specific survival (using dynamic longitudinal information up to 24 months (**Bottom left**) and 12 months (**Bottom right**)). A lower Brier score indicates superior discriminative power of the model and similarly lower ICI, E50, and E90 indicate superior calibration (i.e., less difference between observed and predicted probabilities).

On multivariate joint model for OS when the dynamic changes in Hb, NLR, PLR, LMR, and PSA were combined, every 1 g/dL increase in the current value of Hb was associated with significantly superior OS (HR: 0.88; 95% CI: 0.81–0.94; *p* < 0.001) while an increase in current value of NLR by one point (HR: 1.26; 95% CI: 1.11–1.44; *p* < 0.001) and log of the current value of PSA (HR: 1.24; 95% CI: 1.20–1.28; *p* < 0.001) were associated with significantly inferior OS (Table 3). On multivariate joint model for PCSS, dynamic increase in the log of current value of PSA was associated with significantly inferior PCSS (HR: 1.28; 95% CI: 1.23–1.34; *p* < 0.001). Although increase in the current value of Hb (HR: 0.90; 95% CI: 0.83–0.98; *p* = 0.009) and an increase in the current value of NLR (HR: 1.22; 95% CI: 1.04–1.44; *p* = 0.007) was associated with inferior PCSS, this did not reach our prespecified threshold of significance (Table 3). The time-varying Brier scores from the five-fold cross-validated joint model for OS ranged from 0.12 to 0.16 with longitudinal information up to 24 months and 0.11 to 0.16 with longitudinal information up to 12 months (Figure 2). The time-varying Brier scores from the five-fold cross-validated joint model for PCSS ranged from 0.12 to 0.16 and 0.13 to 0.18 with longitudinal information up to 24 months and 12 months, respectively (Figure 2).

On decision curve analysis, using the multivariate joint model combining information on the dynamic change in the trajectory of Hb, NLR, PLR, and LMR up to 24 months, we did not find any notable benefit over a “treat all” strategy for both OS (Supplementary Figure S17) and PCSS (Supplementary Figure S18). In contrast, the multivariate joint model that used information on dynamic trajectory of log of current value of PSA along with Hb, NLR, PLR, LMR up to 24 months, showed superior net benefits from a threshold of probability of approximately 30% and higher for OS (Supplementary Figure S17), while the same multivariate joint model showed superior net benefits from a threshold of probability of about 20% and higher for PCSS (Supplementary Figure S18). Similar findings were seen when we used longitudinal information up to 12 months (Supplementary Figures S19 and S20).

## 5. Discussion

In this secondary analysis of the LATITUDE trial, we found that inter-patient variation in the dynamic trajectory of PSA was a predictor of OS and PCSS. A multivariate joint model that accounted for only dynamic changes in Hb, NLR, PLR, and LMR was not found to be clinically useful over a “treat all” strategy. However, when inter-patient variation in the dynamic trajectory of PSA was also included along with that of Hb, NLR, LMR, and PLR, it showed some net benefit for both OS and PCSS. This underscores how treatment-induced dynamic change in PSA over time could be used to prognosticate outcome on an individualized basis in mHSPC patients.sro.

Prior studies have shown the association of PSA, either baseline or post-treatment, with survival in mHSPC at various arbitrary timepoints or using various PSA thresholds which could affect reliability or validity of these thresholds [13,14,16]. In contrast, we captured the association of dynamic change in PSA over time without using any arbitrary timepoint or threshold. Further, we demonstrated clinical utility using a net benefit approach. Despite the added complexity in the interpretation of the joint models, this approach is preferable and more useful for individualized prediction of outcome compared to standard predictions from the Cox proportional hazard models which usually apply at the group level to those who share common values of the covariates [36].

Based on recently published clinical trials and meta-analyses, initial management mHSPC usually consists of the doublet of ADT and androgen receptor pathway inhibitors (ARPI) with or without docetaxel [3,4,37,38]. However, the triplet strategy might represent overtreatment in a select group of patients [3,4,7,39]. Our multivariate joint model that comprised dynamic changes in PSA along with other laboratory-based markers showed net benefits at a threshold which is similar to the magnitude of relative survival benefit with triplet strategy in high risk or high volume mHSPC [4,40]. The dynamic trajectory of these serological markers could be a reflection of treatment-induced changes in the overall disease process. Therefore, using the joint model approach, one could identify individuals who are likely to have unfavorable outcome with their ongoing treatment early in the disease course and could utilize this information for sequential treatment intensification for these patients. This hypothesis, if validated, could potentially pave the way for future adaptive trial designs. Our joint model approach should be contrasted to baseline prognostic models in mHSPC which could only predict outcome based on baseline factors but fails to determine how the outcome would fluctuate depending on time- and treatment-dependent variation in the serological markers. This underlying difference between the two approaches is paramount when balancing the benefit and burden of treatment in the evolving disease process of mHSPC.

Our findings, while promising, are not without limitations, including those inherent in any unplanned secondary analysis. The findings are based on a trial cohort of high risk de novo mHSPC and need validation in patients with recurrent mHSPC, patients with low risk mHSPC, and in patients who are managed outside of the clinical trial setting. An additional major limitation for this study is missing information on longitudinal data which may not be missing at random. Use of prior ADT could have influenced the performance status, PSA, and Hb at baseline for some patients. Due to significant computational challenges which are beyond our control and a lack of longitudinal data, we could not combine the dynamic trajectory of additional biomarkers such as serum lactate dehydrogenase or alkaline phosphatase or quality of life parameters such as pain. The findings of the decision curve analyses should be interpreted cautiously due to possible overfitting and for patients who would opt for treatment intensification at a lower threshold of benefit.

## 6. Conclusions

Our study shows that dynamic trajectory of PSA could be used for individualized prognostication of OS and PCSS in high risk de novo mHSPC patients. Our multivariate joint model that included information on dynamic trajectory of PSA along with Hb, NLR, LMR, and PLR could be used to identify individuals who are likely to have unfavorable outcome early in the disease course. If validated in future studies, this joint model approach could aid in designing future adaptive trials investigating sequential treatment personalization based on dynamic trajectory of these five biomarkers for individual patients.

## Figures and Tables

**Table 1:** Baseline characteristics.

	Abiraterone Acetate Plus ADT	ADT Plus Placebo	*p*-Value
n (Total number)	563	575	
Age (median [IQR])	65.0 [60.0, 70.0]	65.0 [60.0, 70.0]	0.44
Age group (%)			
<65	210 (37.3)	220 (38.3)	
65–69	104 (18.5)	132 (23.0)	
70–74	135 (24.0)	108 (18.8)	
≥75	114 (20.2)	115 (20.0)	
Presence of liver metastasis (%)	29 (5.2)	29 (5.0)	0.99
Presence of lung metastasis (%)	70 (12.4)	69 (12.0)	0.89
Nodal stage			
N0/Nx (%)	300 (53.3)	308 (53.6)	0.97
N1 (%)	263 (46.7)	267 (46.4)	
Gleason score			
<9 (%)	263 (46.7)	284 (49.4)	0.40
9–10 (%)	300 (53.3)	291 (50.6)	
ECOG performance status			
0 (%)	313 (55.6)	314 (54.8)	0.83
≥1 (%)	250 (44.4)	260 (45.2)	
Number of skeletal metastases			
0–9	193 (34.3)	201 (35.0)	0.86
≥10 (%)	370 (65.7)	374 (65.0)	
Worst pain score (median (IQR))	1.0 (0.0, 4.0)	1.0 (0.0, 4.0)	0.66
Baseline PSA (median (IQR))	18.4 (3.7, 77.0)	14.8 (2.9, 76.2)	0.20
Baseline Hb (g/dL) (median (IQR))	13.2 (12.0, 14.3)	13.3 (12.1, 14.4)	0.42
Baseline NLR (median (IQR))	2.2 (1.6, 3.0)	2.2 (1.7, 3.0)	0.97
Baseline PLR (median (IQR))	138 (108, 183)	138 (107, 180)	0.93
Baseline LMR (median (IQR))	4.7 (3.6, 6.0)	4.8 (3.6, 6.3)	0.43

NLR: neutrophil to lymphocyte ratio; PLR: platelet to lymphocyte ratio; LMR: lymphocyte to monocyte ratio; Hb: hemoglobin; IQR: inter-quartile range. *p*-values were derived from Chi-square test for categorical variables and Wilcoxon rank-sum test for continuous variables.

**Table 2:** Multivariate joint model summarizing the association of dynamic change in the current level of hemoglobin, neutrophil to lymphocyte ratio, platelet to lymphocyte ratio, and lymphocyte to monocyte ratio together with overall survival (OS) and prostate cancer-specific survival (PCSS).

Models	Parameters	Hazard Ratio	Lower CI	Upper CI	*p*-Value
For OS	ADT alone vs. Abiraterone plus ADT	1.61	1.28	2.04	***<0.001***
	Skeletal lesions (10 or more vs. 0–10)	1.69	1.38	2.06	***<0.001***
	ECOG performance status (1–2 vs. 0)	1.31	1.11	1.56	0.004
	Nodal stage	1.08	0.92	1.28	0.35
	Liver metastasis (yes vs. no)	1.56	1.10	2.16	0.008
	Gleason score (9–10 vs. <9)	1.22	1.03	1.45	0.02
	Baseline worst pain score	1.05	1.02	1.09	***0.003***
	Baseline Hb	0.99	0.99	1.00	0.01
	Log of baseline PSA	0.96	0.93	1.01	0.08
	Dynamic change in the current Hb level by 1 g/dL	0.80	0.75	0.86	***<0.001***
	Dynamic change in the current NLR level by 1 point	1.19	1.06	1.33	***0.003***
	Dynamic change in the current PLR level by 100 points	1.07	0.87	1.31	0.47
	Dynamic change in the current LMR by 5 points	0.90	0.68	1.10	0.43
For PCSS	ADT alone vs. Abiraterone plus ADT	1.71	1.32	2.25	***<0.001***
	Skeletal lesions (10 or more vs. 0–10)	1.88	1.49	2.41	***<0.001***
	ECOG performance status (1–2 vs. 0)	1.30	1.08	1.58	0.008
	Nodal stage	1.03	0.85	1.25	0.80
	Liver metastasis (yes vs. no)	1.69	1.14	2.43	0.008
	Gleason score (9–10 vs. <9)	1.27	1.05	1.54	0.02
	Baseline worst pain score	1.05	1.01	1.09	0.02
	Baseline Hb	0.99	0.98	1.00	0.006
	Log of baseline PSA	0.97	0.93	1.01	0.15
	Dynamic change in the current Hb level by 1 g/dL	0.81	0.75	0.87	***<0.001***
	Dynamic change in the current NLR level by 1 point	1.11	0.97	1.27	0.13
	Dynamic change in the current PLR level by 100 points	1.19	0.93	1.51	0.17
	Dynamic change in the current LMR by 5 points	0.86	0.61	1.10	0.37

NLR: neutrophil to lymphocyte ratio; PLR: platelet to lymphocyte ratio; LMR: lymphocyte to monocyte ratio; Hb: hemoglobin. Bold and italicized *p*-values indicate statistical significance.

**Table 3:** Multivariate joint model summarizing the association of dynamic change in the current level of hemoglobin, neutrophil to lymphocyte ratio, platelet to lymphocyte ratio, lymphocyte to monocyte ratio, and prostate-specific antigen together with overall survival (OS) and prostate cancer-specific survival (PCSS).

Models	Parameters	Hazard Ratio	Lower CI	Upper CI	*p*-Value
For OS	ADT alone vs. abiraterone plus ADT	0.87	0.66	1.13	0.29
	Skeletal lesions (10 or more vs. 0–10)	1.34	1.07	1.68	0.01
	ECOG performance status (1–2 vs. 0)	1.33	1.11	1.58	0.004
	Nodal stage	1.14	0.96	1.36	0.16
	Liver metastasis (yes vs. no)	1.78	1.23	2.49	***0.001***
	Gleason score (9–10 vs. <9)	1.17	0.98	1.38	0.07
	Baseline worst pain score	1.04	1.01	1.09	0.02
	Baseline Hb	0.99	0.99	1.00	***0.001***
	Log of baseline PSA	0.92	0.88	0.96	***<0.001***
	Dynamic change in the current Hb level by 1 g/dL	0.88	0.81	0.94	***<0.001***
	Dynamic change in the current NLR level by 1 point	1.26	1.11	1.44	***<0.001***
	Dynamic change in the current PLR level by 100 points	0.97	0.76	1.22	0.79
	Dynamic change in the current LMR by 5 points	1.02	0.78	1.22	0.81
	Dynamic change in the log of current PSA value	1.24	1.20	1.28	***<0.001***
For PCSS	ADT alone vs. abiraterone plus ADT	0.82	0.59	1.15	0.24
	Skeletal lesions (10 or more vs. 0–10)	1.39	1.07	1.82	0.02
	ECOG Performance status (1–2 vs. 0)	1.29	1.04	1.58	0.02
	Nodal stage	1.11	0.91	1.35	0.30
	Liver metastasis (yes vs. no)	1.99	1.33	2.89	***<0.001***
	Gleason score (9–10 vs. <9)	1.21	1.00	1.51	0.05
	Baseline worst pain score	1.04	1.00	1.09	0.05
	Baseline Hb	0.99	0.98	1.00	***0.001***
	Log of baseline PSA	0.92	0.88	0.97	***0.001***
	Dynamic change in the current Hb level by 1 g/dL	0.90	0.83	0.98	0.009
	Dynamic change in the current NLR level by 1 point	1.22	1.04	1.44	0.007
	Dynamic change in the current PLR level by 100 points	1.01	0.76	1.32	0.95
	Dynamic change in the current LMR by 5 points	0.98	0.68	1.23	0.99
	Dynamic change in the log of current PSA value	1.28	1.23	1.34	***<0.001***

NLR: neutrophil to lymphocyte ratio; PLR: platelet to lymphocyte ratio; LMR: lymphocyte to monocyte ratio; Hb: hemoglobin; PSA: prostate-specific antigen. Bold and italicized *p*-values indicate statistical significance.

## Data Availability

This study, carried out under YODA Project # 2022-4566, used data obtained from the Yale University Open Data Access Project, which has an agreement with JANSSEN RESEARCH & DEVELOPMENT, L.L.C. The interpretation and reporting of research using this data are solely the responsibility of the authors and does not necessarily represent the official views of the Yale University Open Data Access Project or JANSSEN RESEARCH & DEVELOPMENT, L.L.C.

## Supplementary Materials

The following supporting information can be downloaded at: https://www.mdpi.com/article/10.3390/cancers15174392/s1, Supplementary Methods: Decision curve analysis of multivariate joint models; Figure S1: Calibration indices and time-varying Brier scores for univariate joint model determining the association of dynamic change in Hb level with PCSS (capturing longitudinal information up to 24 months and determining survival up to 60 months); Figure S2: Calibration indices and time-varying Brier scores for univariate joint model determining the association of dynamic change in Hb level with OS (capturing longitudinal information up to 24 months and determining survival up to 60 months); Figure S3: Calibration indices and time-varying Brier scores for univariate joint model determining the association of dynamic change in Hb level with PCSS (capturing longitudinal information up to 12 months and determining survival up to 60 months); Figure S4: Calibration indices and time-varying Brier scores for univariate joint model determining the association of dynamic change in Hb level with OS (capturing longitudinal information up to 12 months and determining survival up to 60 months); Figure S5: Calibration indices and time-varying Brier scores for univariate joint model determining the association of dynamic change in NLR level with PCSS (capturing longitudinal information up to 24 months and determining survival up to 60 months); Figure S6: Calibration indices and time-varying Brier scores for univariate joint model determining the association of dynamic change in NLR level with OS (capturing longitudinal information up to 24 months and determining survival up to 60 months); Supplementary Figure S7: Calibration indices and time-varying Brier scores for univariate joint model determining the association of dynamic change in NLR level with PCSS (capturing longitudinal information up to 12 months and determining survival up to 60 months); Figure S8: Calibration indices and time-varying Brier scores for univariate joint model determining the association of dynamic change in NLR level with OS (capturing longitudinal information up to 12 months and determining survival up to 60 months); Figure S9: Calibration indices and time-varying Brier scores for univariate joint model determining the association of dynamic change in PLR level with PCSS (capturing longitudinal information up to 24 months and determining survival up to 60 months); Figure S10: Calibration indices and time-varying Brier scores for univariate joint model determining the association of dynamic change in PLR level with OS (capturing longitudinal information up to 24 months and determining survival up to 60 months); Figure S11: Calibration indices and time-varying Brier scores for univariate joint model determining the association of dynamic change in PLR level with PCSS (capturing longitudinal information up to 12 months and determining survival up to 60 months); Figure S12: Calibration indices and time-varying Brier scores for univariate joint model determining the association of dynamic change in PLR level with OS (capturing longitudinal information up to 12 months and determining survival up to 60 months); Figure S13: Calibration indices and time-varying Brier scores for univariate joint model determining the association of dynamic change in LMR level with PCSS (capturing longitudinal information up to 24 months and determining survival up to 60 months); Figure S14: Calibration indices and time-varying Brier scores for univariate joint model determining the association of dynamic change in LMR level with OS (capturing longitudinal information up to 24 months and determining survival up to 60 months); Figure S15: Calibration indices and time-varying Brier scores for univariate joint model determining the association of dynamic change in LMR level with PCSS (capturing longitudinal information up to 12 months and determining survival up to 60 months); Figure S16: Calibration indices and time-varying Brier scores for univariate joint model determining the association of dynamic change in LMR level with OS (capturing longitudinal information up to 12 months and determining survival up to 60 months); Figure S17: Decision curve analysis plot for multivariate joint models (with and without PSA) for OS using longitudinal information up to a landmark time of 24 months; Figure S18: Decision curve analysis plot for multivariate joint models (with and without PSA) for PCSS using longitudinal information up to a landmark time of 24 months; Figure S19: Decision curve analysis plot for multivariate joint models (with and without PSA) for OS using longitudinal information up to a landmark time of 12 months; Figure S20: Decision curve analysis plot for multivariate joint models (with and without PSA) for PCSS using longitudinal information up to a landmark time of 12 months.
