# Supplementary material for: Prognostic Role of Dynamic Changes in Serological Markers in Metastatic Hormone Naïve Prostate Cancer
Source: Cancers (Basel). 2023 Sep 2;15(17):4392. doi: 10.3390/cancers15174392 (PMC10486494; doi:10.3390/cancers15174392)

Supplementary Material

Supplementary Methods:

Decision curve analysis of multivariate joint models

To apply the decision curve analysis in the context of multivariate joint models for OS, we initially captured longitudinal information for the serological markers up to a landmark time of 24 months. Subsequently, we applied the joint model to determine the predicted cumulative incidence of deaths for the individuals. A decision curve analysis was performed to determine the net benefit of applying the multivariate joint models across various threshold probabilities and the decision curve was generated plotting the net benefits against a range of threshold probabilities. This method has been clearly summarized by Vickers et al [35]. This includes results for “intervention for all” and “intervention for none”. Given treatments were combined and events by weighting the proportion of treatments by the ratio of harm from treatment and harm from event. This was Similar decision curve analysis was performed with longitudinal information for the serological markers up to a landmark time of 12 months.

Supplementary Figures

Figure S1: Calibration indices and time-varying Brier scores for univariate joint model determining the association of dynamic change in Hb level with PCSS (capturing longitudinal information up to 24 months and determining survival up to 60 months)

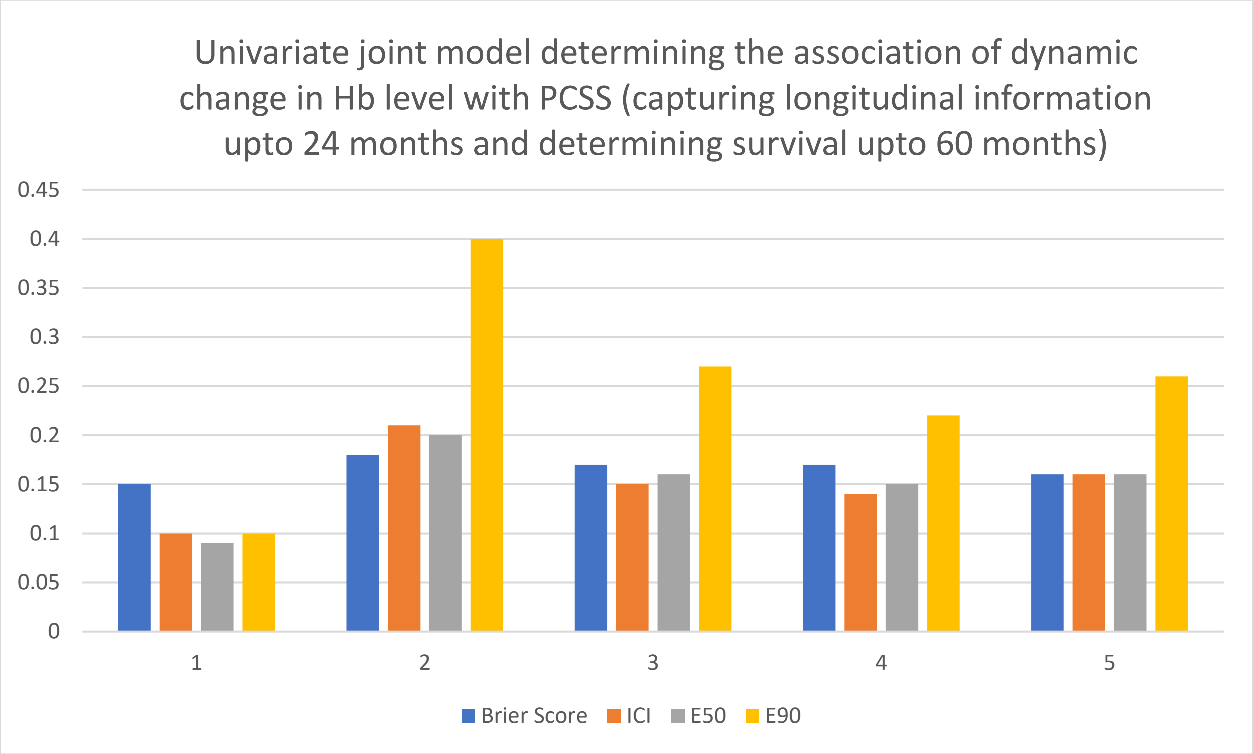

Figure S2: Calibration indices and time-varying Brier scores for univariate joint model determining the association of dynamic change in Hb level with OS (capturing longitudinal information up to 24 months and determining survival up to 60 months)

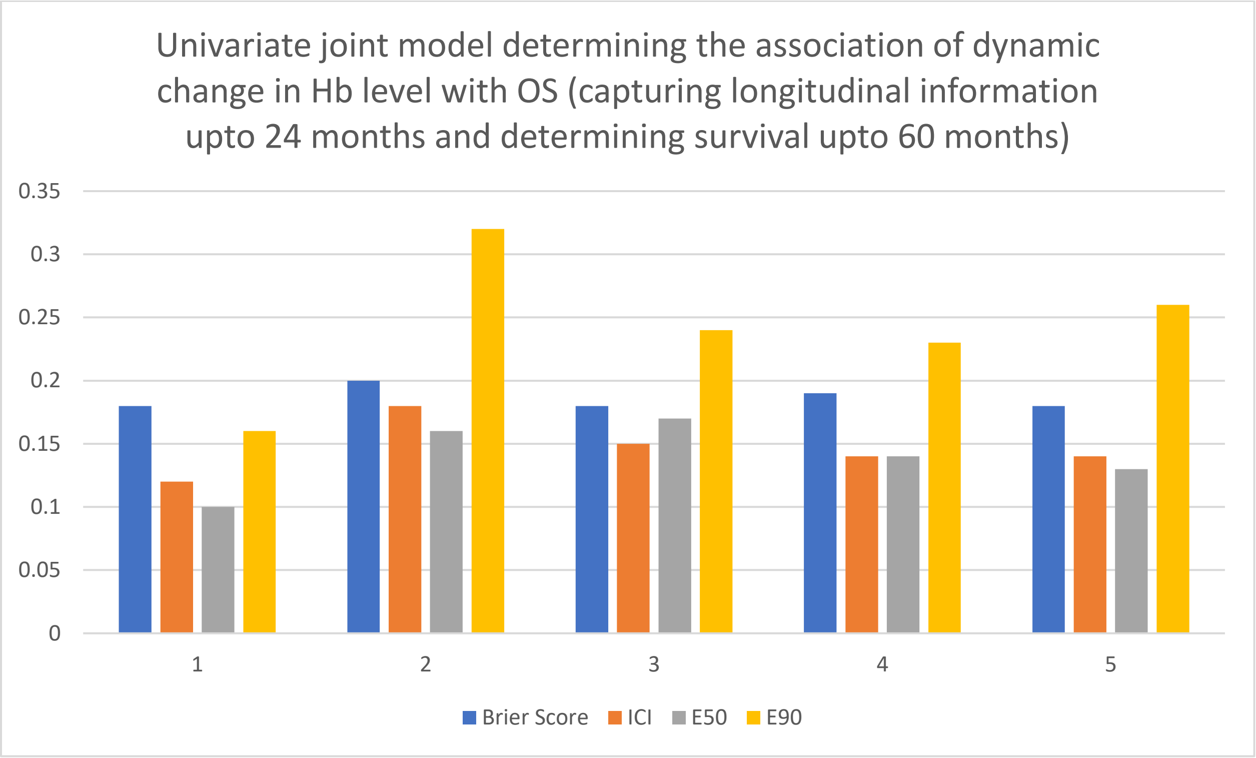

Figure S3: Calibration indices and time-varying Brier scores for univariate joint model determining the association of dynamic change in Hb level with PCSS (capturing longitudinal information up to 12 months and determining survival up to 60 months)

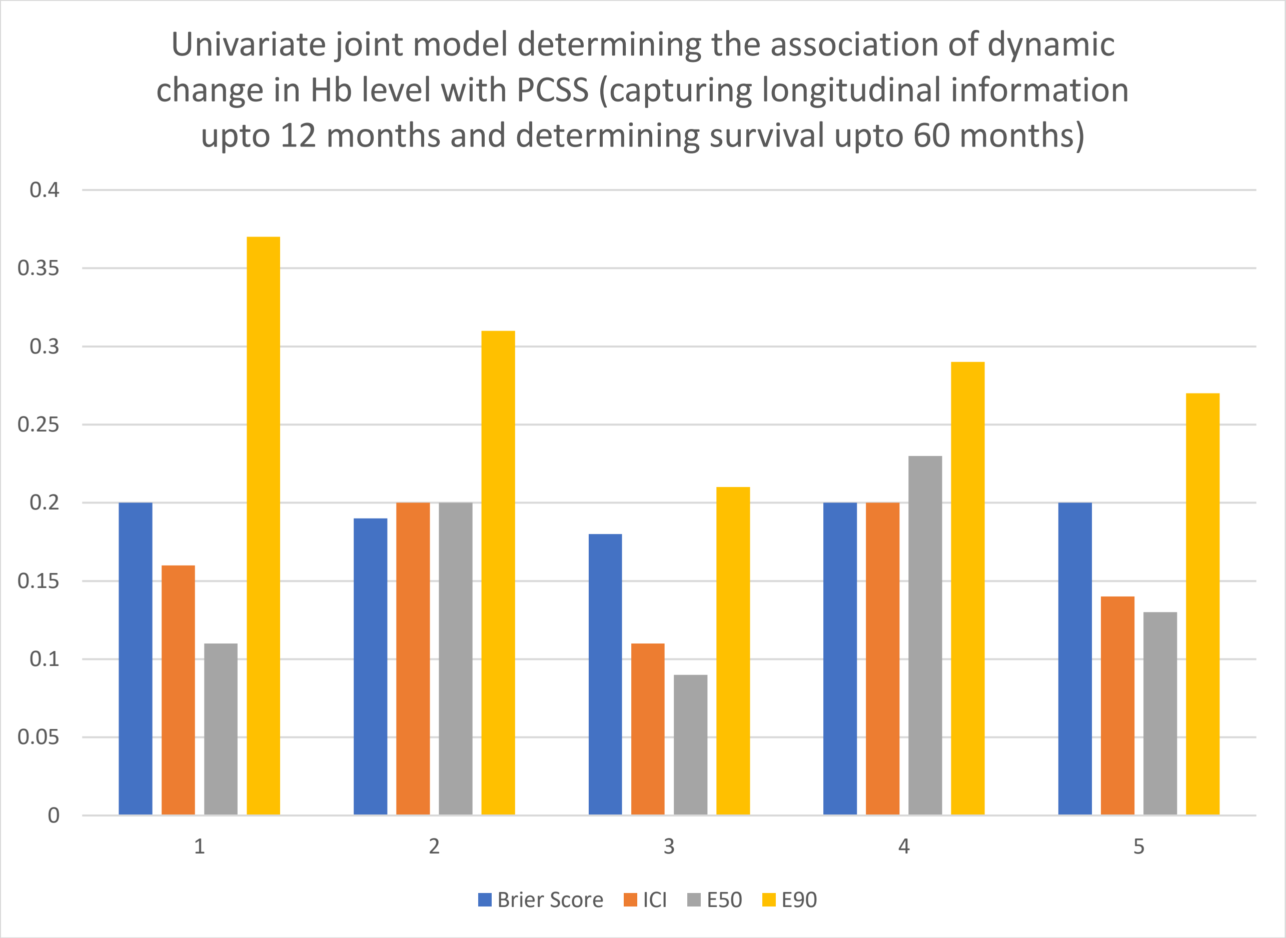

Figure S4: Calibration indices and time-varying Brier scores for univariate joint model determining the association of dynamic change in Hb level with OS (capturing longitudinal information up to 12 months and determining survival up to 60 months)

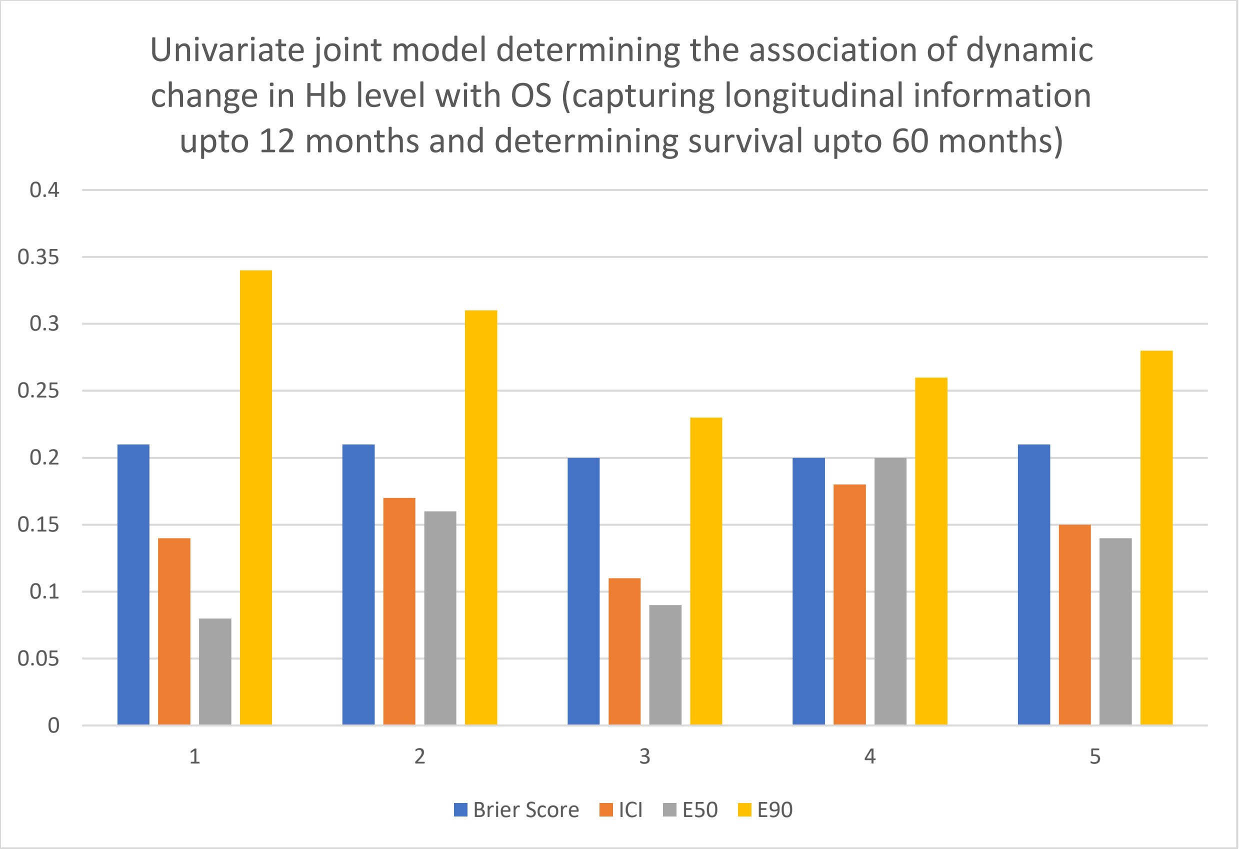

Figure S5: Calibration indices and time-varying Brier scores for univariate joint model determining the association of dynamic change in NLR level with PCSS (capturing longitudinal information up to 24 months and determining survival up to 60 months)

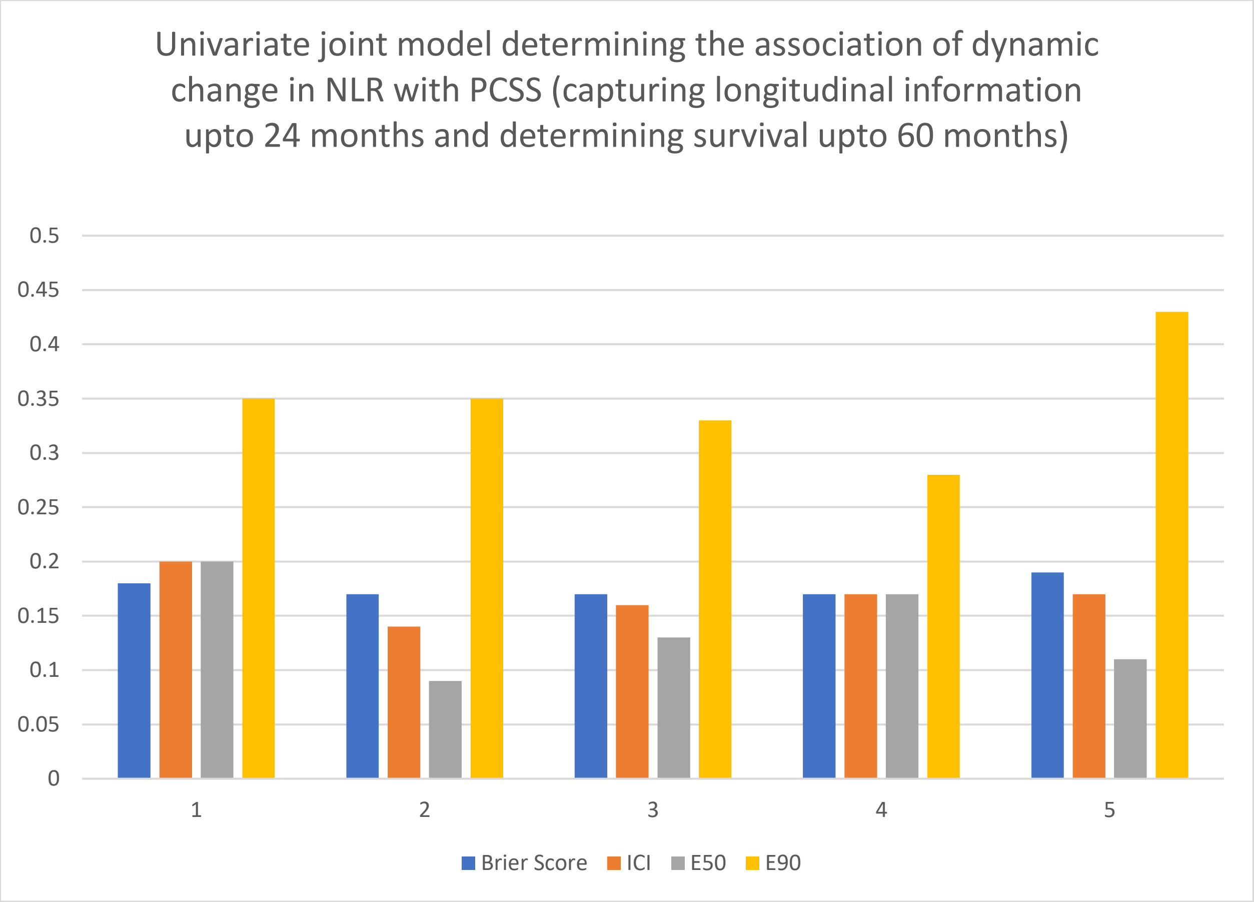

Figure S6: Calibration indices and time-varying Brier scores for univariate joint model determining the association of dynamic change in NLR level with OS (capturing longitudinal information up to 24 months and determining survival up to 60 months)

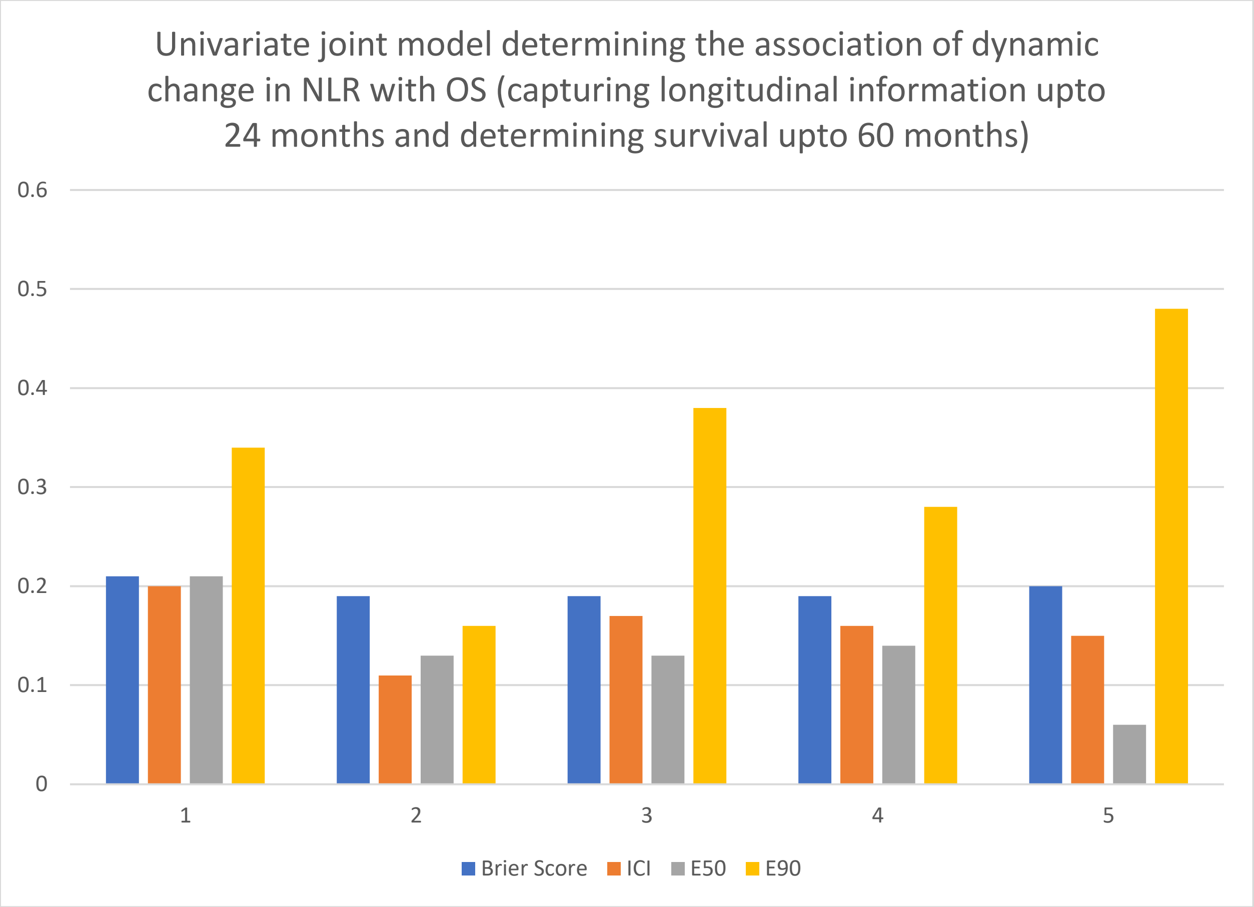

Figure S7: Calibration indices and time-varying Brier scores for univariate joint model determining the association of dynamic change in NLR level with PCSS (capturing longitudinal information up to 12 months and determining survival up to 60 months)

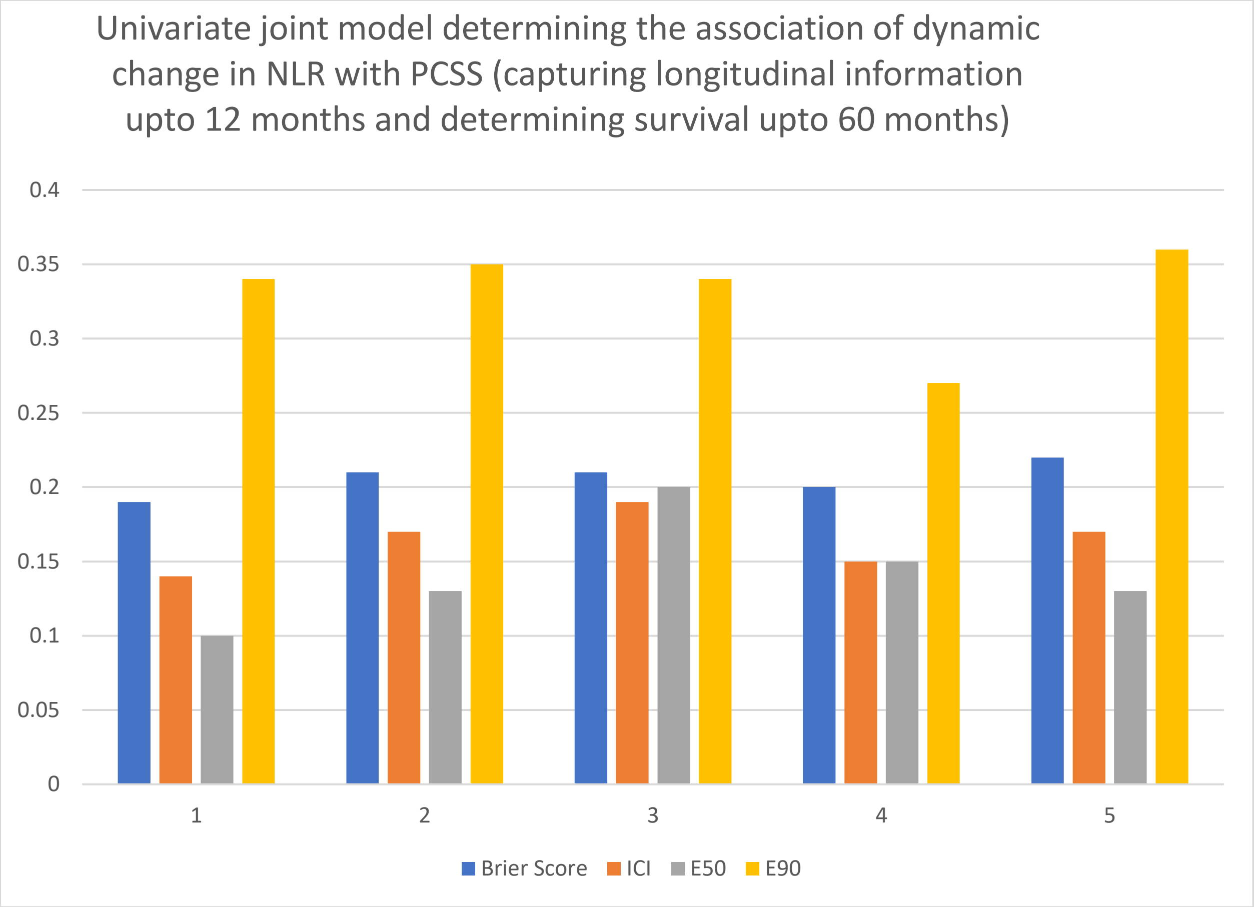

Figure S8: Calibration indices and time-varying Brier scores for univariate joint model determining the association of dynamic change in NLR level with OS (capturing longitudinal information up to 12 months and determining survival up to 60 months)

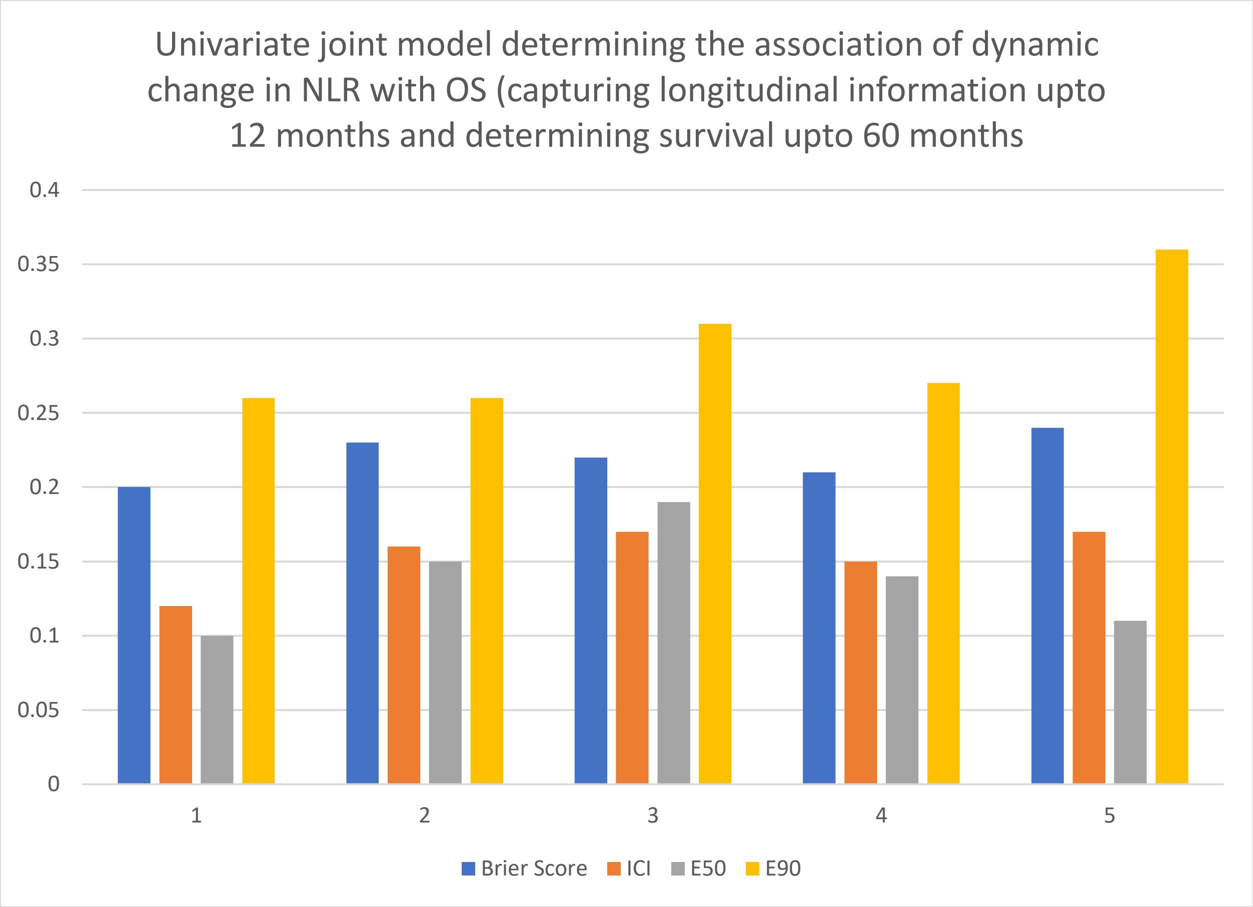

Figure S9: Calibration indices and time-varying Brier scores for univariate joint model determining the association of dynamic change in PLR level with PCSS (capturing longitudinal information up to 24 months and determining survival up to 60 months)

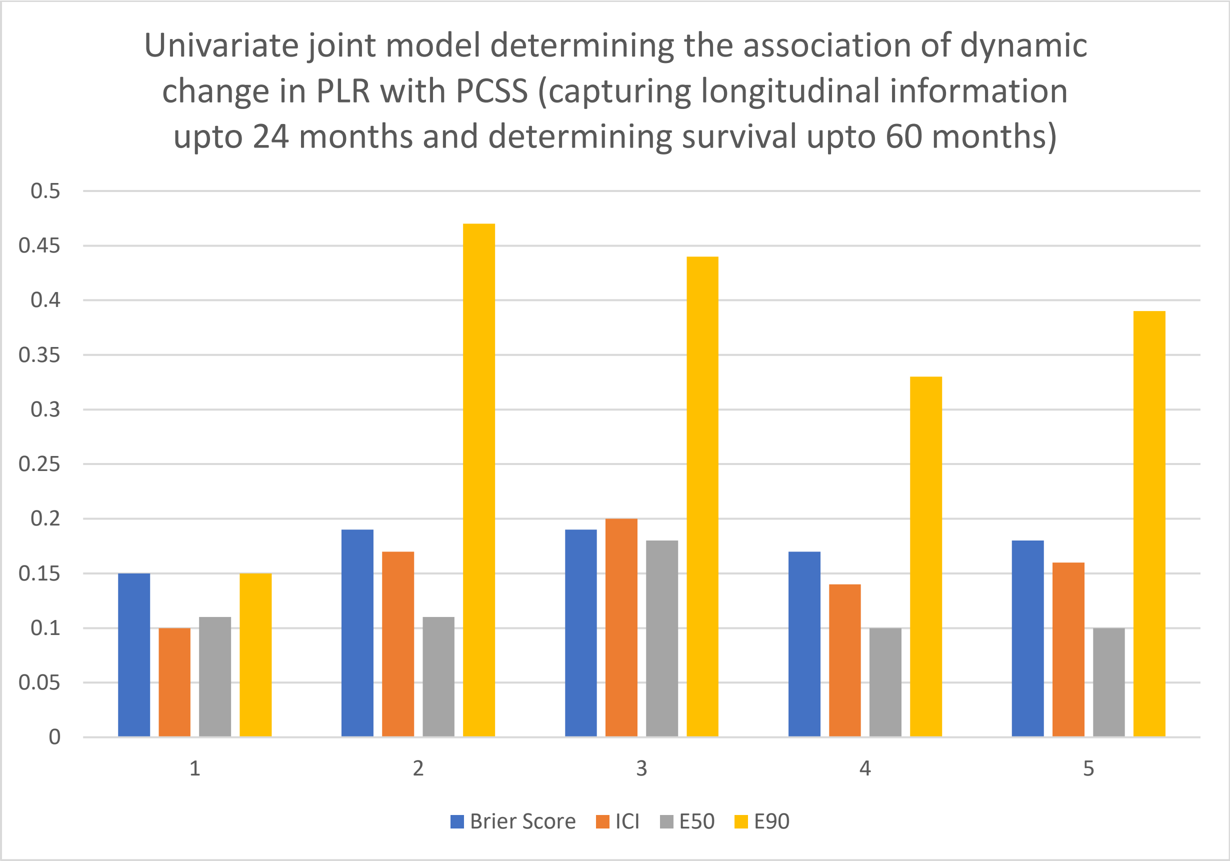

Figure S10: Calibration indices and time-varying Brier scores for univariate joint model determining the association of dynamic change in PLR level with OS (capturing longitudinal information up to 24 months and determining survival up to 60 months)

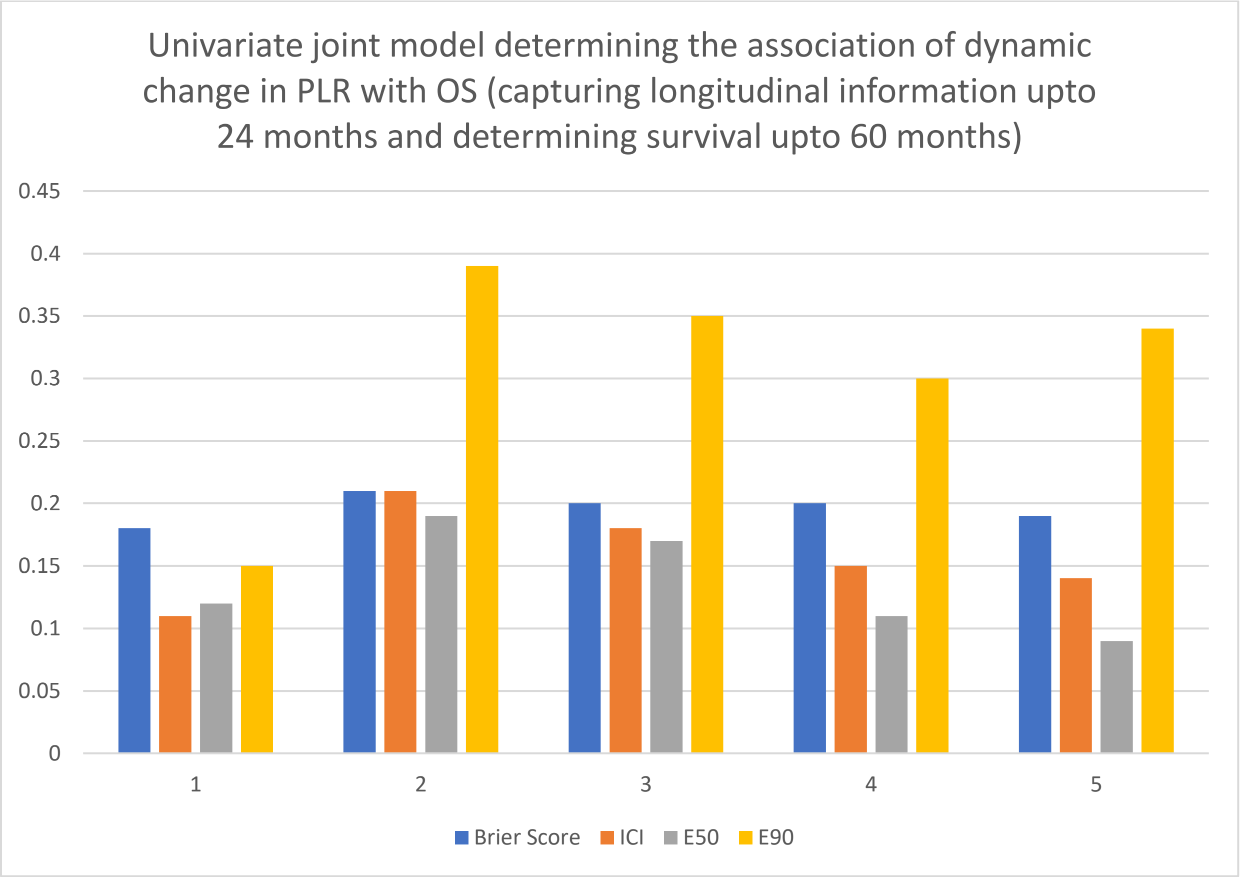

Figure S11: Calibration indices and time-varying Brier scores for univariate joint model determining the association of dynamic change in PLR level with PCSS (capturing longitudinal information up to 12 months and determining survival up to 60 months)

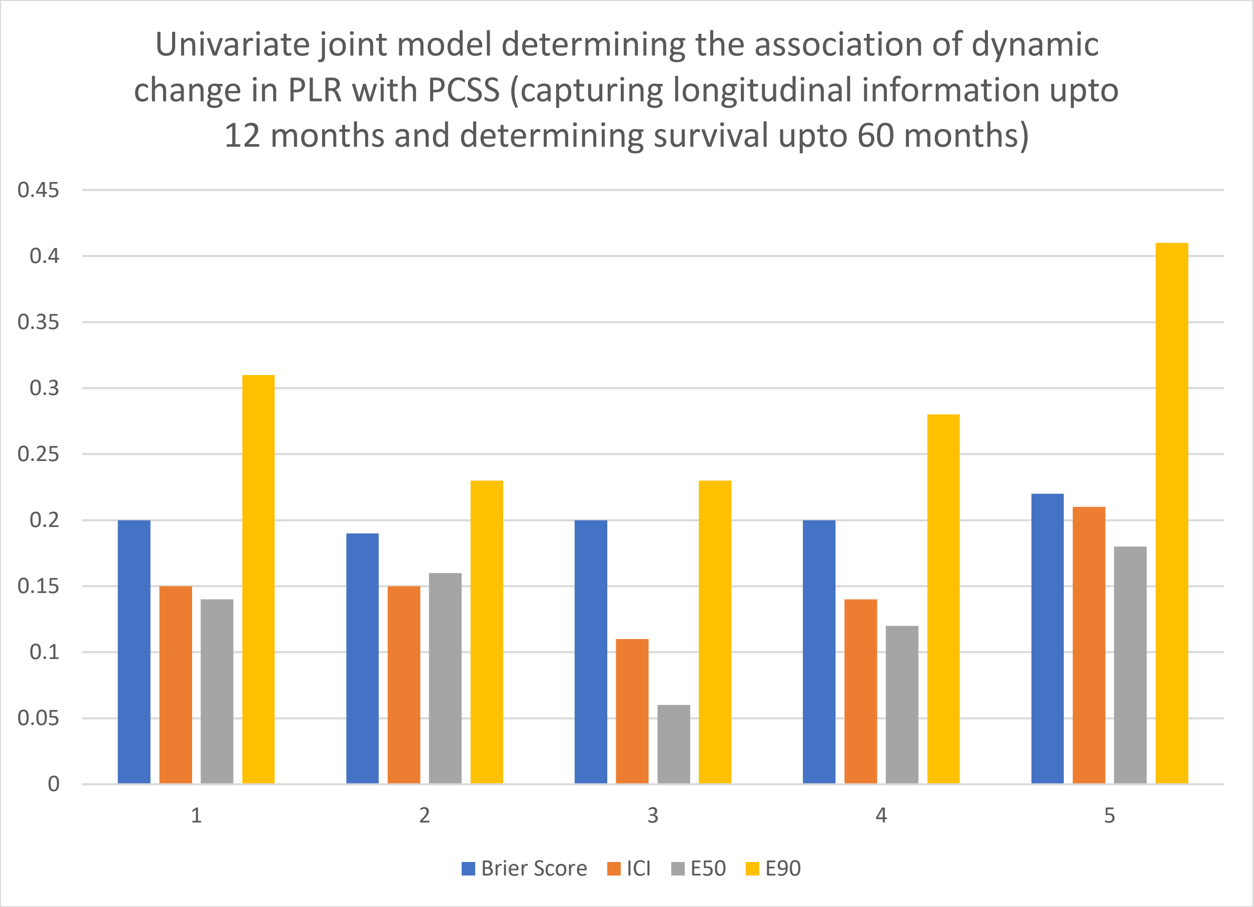

Figure S12: Calibration indices and time-varying Brier scores for univariate joint model determining the association of dynamic change in PLR level with OS (capturing longitudinal information up to 12 months and determining survival up to 60 months)

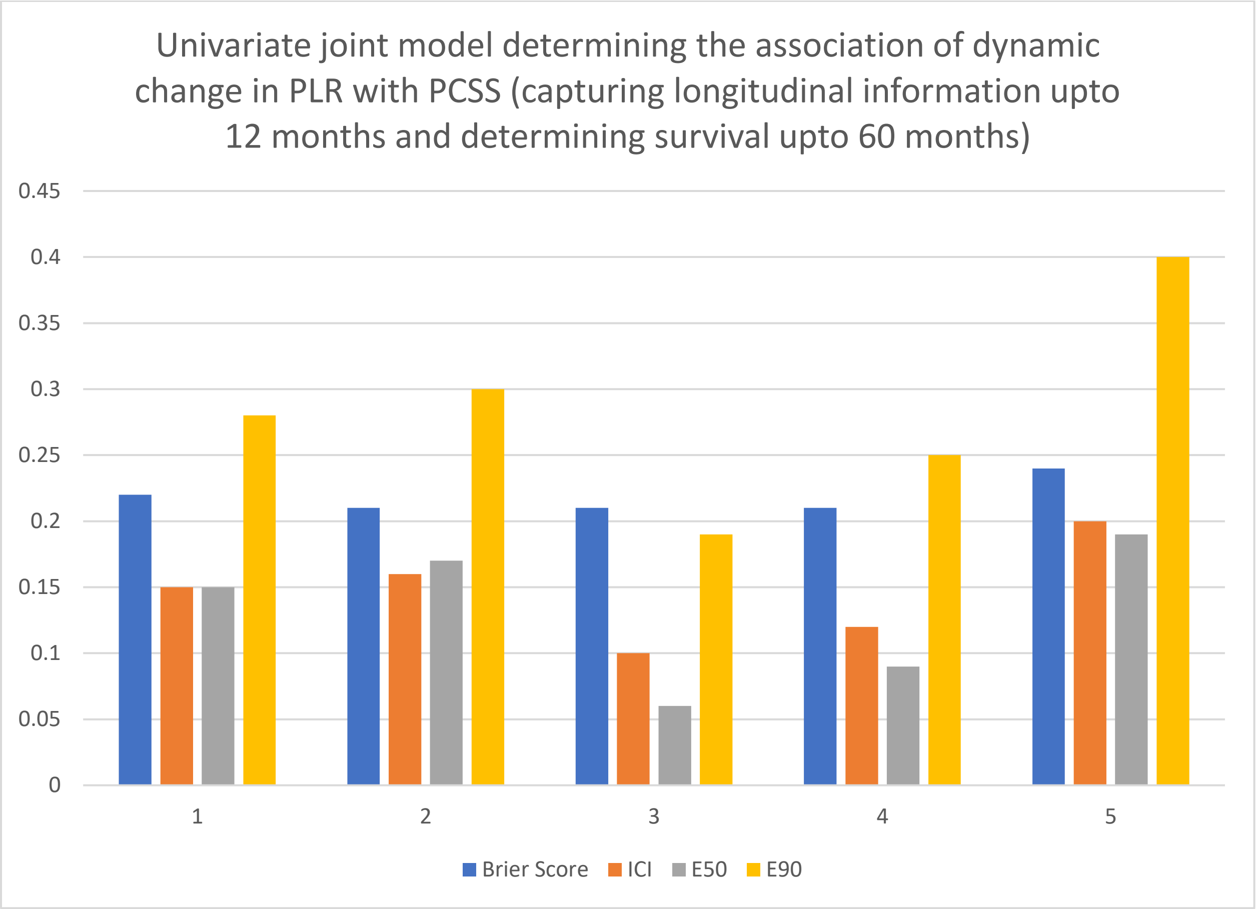

Figure S13: Calibration indices and time-varying Brier scores for univariate joint model determining the association of dynamic change in LMR level with PCSS (capturing longitudinal information up to 24 months and determining survival up to 60 months)

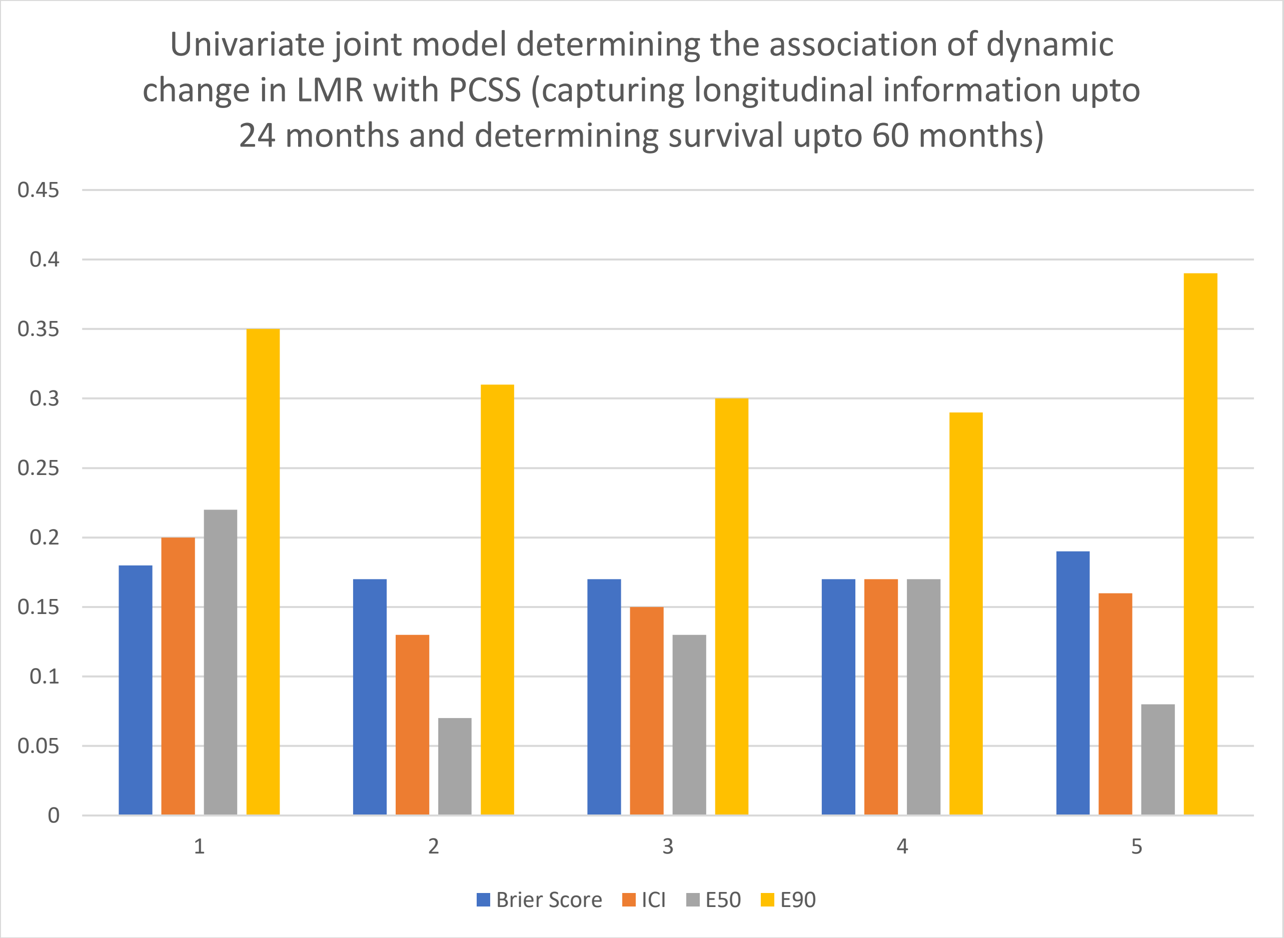

Figure S14: Calibration indices and time-varying Brier scores for univariate joint model determining the association of dynamic change in LMR level with OS (capturing longitudinal information up to 24 months and determining survival up to 60 months)

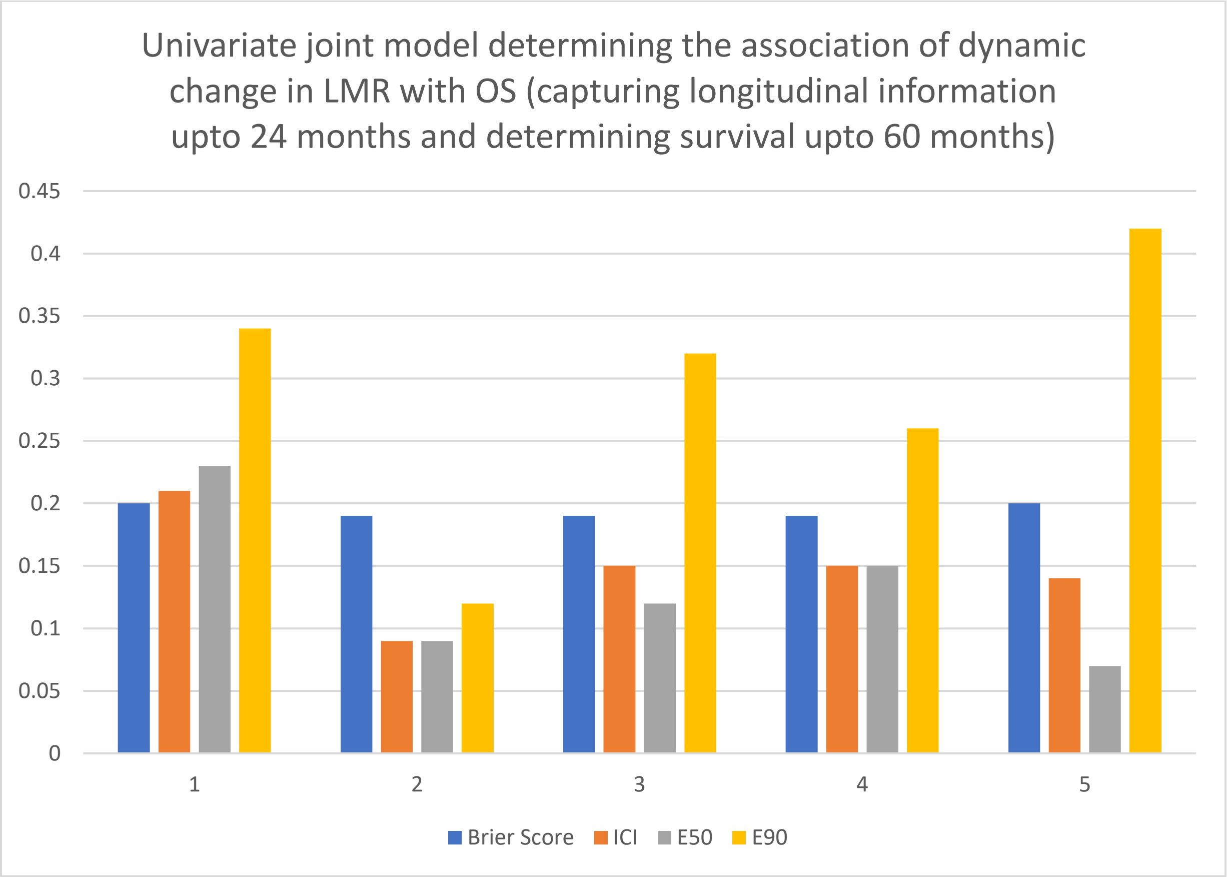

Figure S15: Calibration indices and time-varying Brier scores for univariate joint model determining the association of dynamic change in LMR level with PCSS (capturing longitudinal information up to 12 months and determining survival up to 60 months)

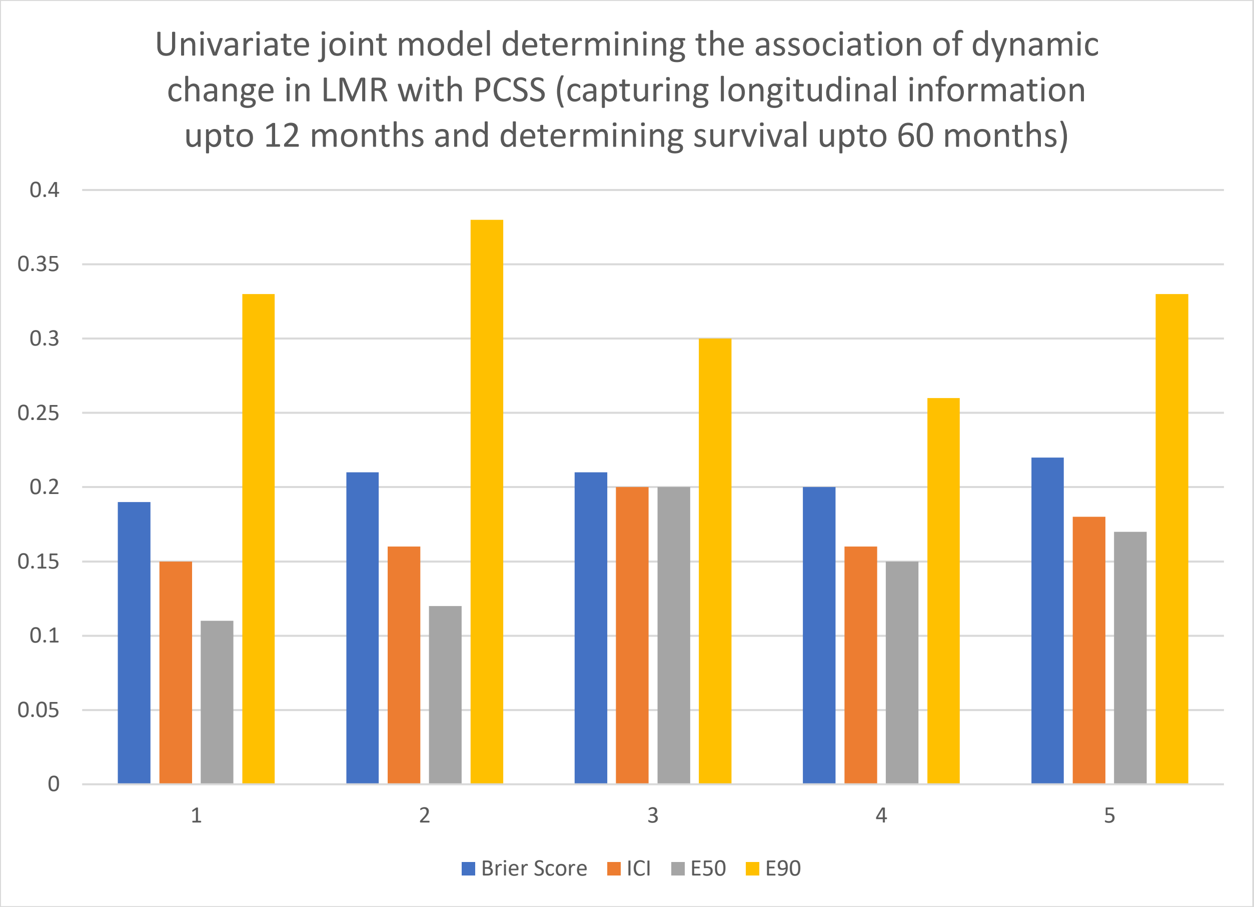

Figure S16: Calibration indices and time-varying Brier scores for univariate joint model determining the association of dynamic change in LMR level with OS (capturing longitudinal information up to 12 months and determining survival up to 60 months)

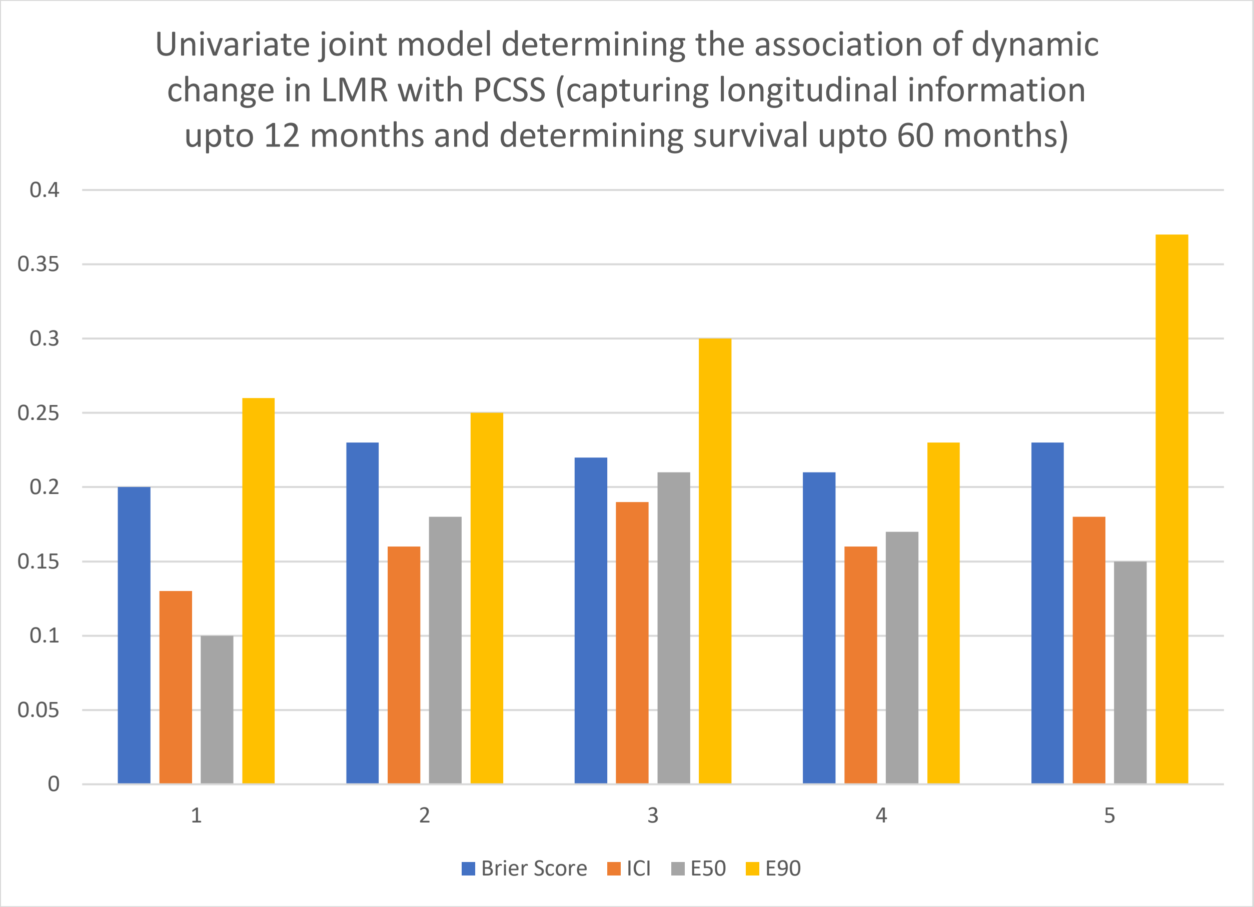

Figure S17: Decision curve analysis plot for multivariate joint models (with and without PSA) for OS using longitudinal information up to a landmark time of 24 months.

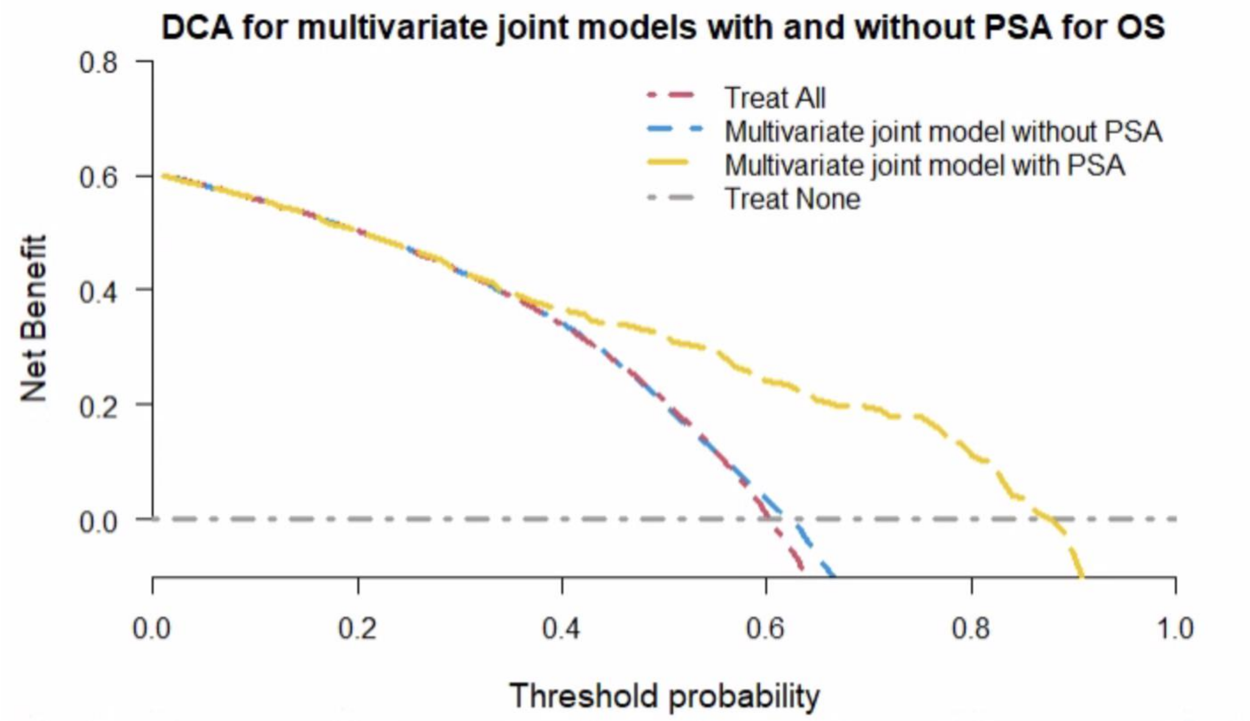

Figure S18: Decision curve analysis plot for multivariate joint models (with and without PSA) for PCSS using longitudinal information up to a landmark time of 24 months.

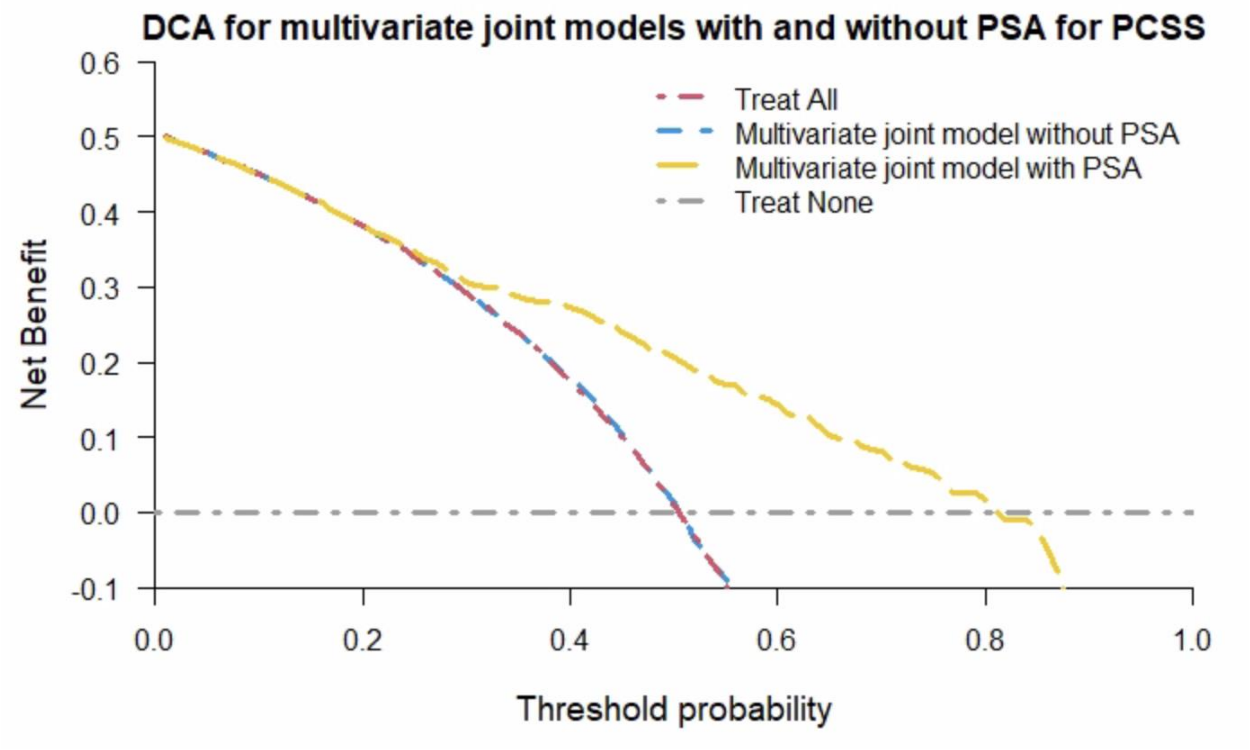

Figure S19: Decision curve analysis plot for multivariate joint models (with and without PSA) for OS using longitudinal information up to a landmark time of 12 months.

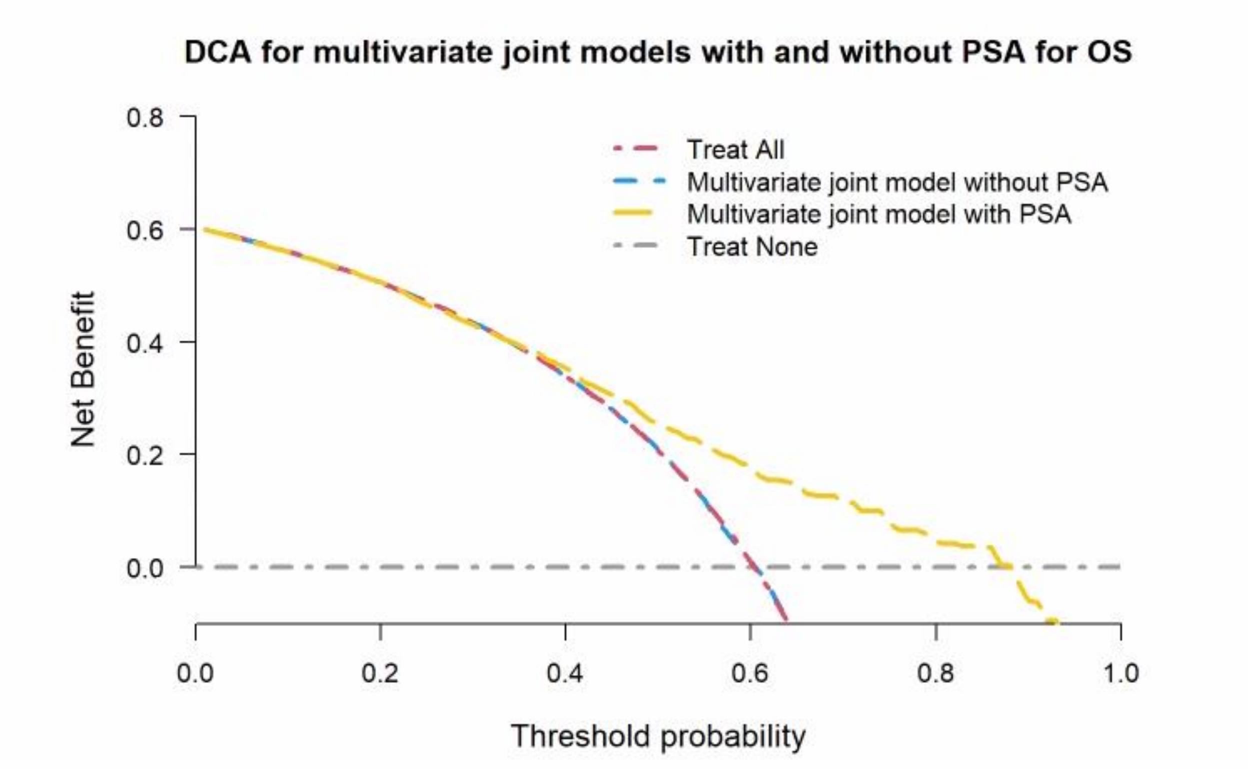

Figure S20: Decision curve analysis plot for multivariate joint models (with and without PSA) for PCSS using longitudinal information up to a landmark time of 12 months.

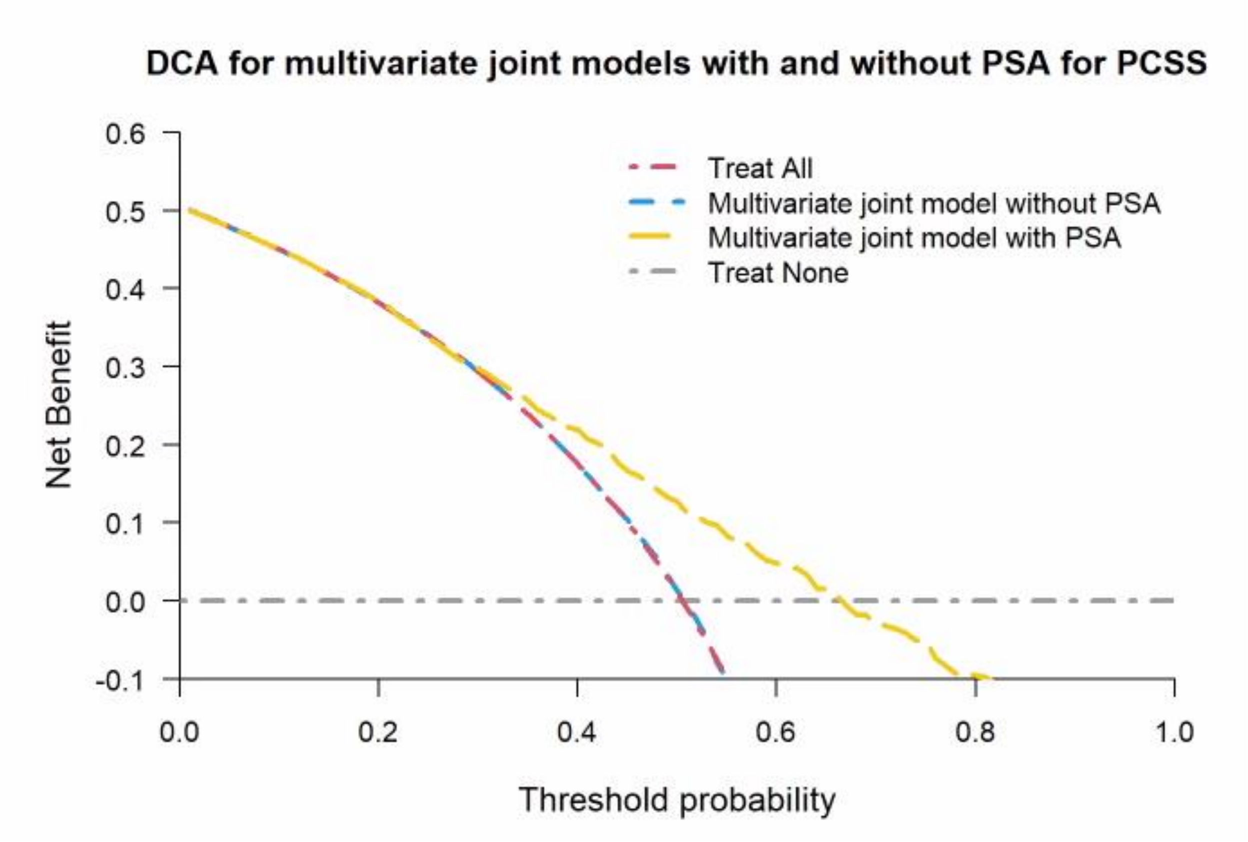

Supplement: Supplementary file 1 [file cancers-15-04392-s001.zip › cancers-2553072-supplementary.pdf]
